# Glycan-related genes in human gut microbiota exhibit differential distribution and diversity in carbohydrate degradation and glycan synthesis

**DOI:** 10.3389/fmolb.2023.1137303

**Published:** 2023-06-15

**Authors:** Hayato Takihara, Shujiro Okuda

**Affiliations:** Medical AI Center, Niigata University School of Medicine, Niigata, Niigata, Japan

**Keywords:** glycan-related genes/CAZymes, metagenome, human gut microbiome, carbohydrates/glycan, glycoside hydrolase, glycosyltransferase (GT)

## Abstract

Interactions between humans and the gut microbiome occur by supplying nutrients to gut epithelial cells via short-chain fatty acids obtained from dietary carbohydrates or mucins and activating immunity via mucins’ degradation. The degradation of carbohydrates derived from food is an important function for organisms to obtain energy. However, since humans possess only 17 genes encoding carbohydrate-degrading enzymes, the gut microbiome is responsible for degrading plant-derived polysaccharides. Using the method for extracting glycan-related genes from the metagenomes constructed thus far, we calculated the distribution and abundance of different glycan-related genes in the healthy human gut metagenome. Glycan-related genes showed an abundance of 0.64–11.00, indicating large individual differences. However, the distribution of the classes of glycan-related genes was similar between the samples. In addition, the function of carbohydrate degradation was divided into three clusters, showing high diversity; however, the synthesis function was not divided, indicating low diversity. The substrates of enzymes for carbohydrate degradation between clusters were either plant-derived polysaccharides or biased toward degrading polysaccharides derived from other sources. These functional biases differ depending on the type of microorganism used. Based on these findings, we predicted that 1) diversity will be constant because the influence on the host by the transferase of gut bacteria is a function derived from the genome, and 2) diversity will be high because the influence on the host by the hydrolase of gut bacteria is affected by incoming dietary carbohydrates.

## 1 Introduction

Organisms can efficiently obtain energy by degrading food-derived carbohydrates. Carbohydrates are a major source of energy and important building blocks of life. Based on their mechanisms of action, the enzymes involved in these metabolic processes are classified as glycoside hydrolases (GHs), carbohydrate esterases (CEs), polysaccharide lyases (PLs), glycosyltransferases (GTs), carbohydrate-binding modules (CBMs), and auxiliary activities (AAs). These six enzymes and gene sequences classes are collectively termed CAZymes (Henrissat, 1991; Henrissat and Bairocht, 1993; Campbell JA, 1997; Lombard et al., 2010). Despite being an important energy source, the human genome encodes only 17 carbohydrate-degrading enzymes. The gut microbiota performs most carbohydrate degradation (Kaoutari et al., 2013). Cresswell et al. (Cresswell et al., 2020) reported that the gut microbiota changed with different types of dietary polysaccharides. This phenomenon is thought to be caused by bacteria with different carbohydrate transporters and polysaccharide-utilizing enzymes that colonize the human gut. Individuals with different glycan-related gene compositions in their gut microbiota may have different metabolic capacities for carbohydrate degradation and absorption. In the Hadza people, whose diet varies greatly depending on the time of year because they hunt differently in rainy and dry seasons, the polysaccharide-degrading genes possessed by gut bacteria differ depending on the season (Smits et al., 2017; Merill et al., 2019).

In contrast, Bhattacharya et al. showed that the ability of gut bacteria to metabolize carbohydrates was regionally classified using their proposed CAZotype, comparing the types and abundance of CAZymes possessed by the human gut microbiota (Bhattacharaya et al., 2015). Additionally, Bhattacharya et al. (Bhattacharya et al., 2015) reported differences in the abundance and types of hydrolases among people of different age groups and body mass indices (BMI). They reported a correlation between BMI and the abundance of GH13 (α-amylase) and correlations among different species possessing GH13 (Bhattacharya et al., 2015). Many genes that degrade seaweed-derived polysaccharides have been identified in the gut microbiome of Japanese individuals (Nishijima et al., 2016). The glycan-related genes possessed by the gut microbiome are thought to be partly due to the individual’s life and partly due to local culture. Although these reports compared the diversity between samples using Shannon index and GINI coefficient, they did not clarify what caused the difference in diversity.

Takihara et al. (2021) developed a method to calculate the abundance of glycan-related genes and the distribution of enzyme functions in the environmental metagenome. We detected glycan-related genes in various environments, glycan-related genes in specific environments, and the relationship between polysaccharides present in the environment and polysaccharide-degrading genes of microorganisms. The abundance of glycan-related genes in the human gut metagenome examined at that time was 3%, which was higher than that in other environments. Although the Shannon index of the metagenomes in the human intestine were similar, the number of samples was small. As the gut environment continuously receives carbohydrates derived from food, the abundance and functional composition of glycan-related genes may respond accordingly. In this study, we examined whether the distribution of the abundance and function of glycan-related genes in the human gut microbiome differs among individuals, investigated 17 types of carbohydrate hydrolases present in humans, and determined their roles.

## 2 Materials and methods

### 2.1 Acquisition of metagenome sequences and metadata

We extracted glycan-related genes from the metagenome of the healthy human gut microbiome. Sequencer type, disease information, and nationality metadata were obtained from the Data Repository For Human Gut Microbiota (Dai et al., 2022). Using the metadata, we searched for metagenomes with a healthy phenotype, 100 bp or more per read, 10 million or more total reads, and MiSeq or HiSeq output sequences. Long-read metagenomic sequences were not included in the analysis because the number of samples could not be determined. From the National Center for Biotechnology Information (NCBI) Sequence Read Archive (SRA) (https://www.ncbi.nlm.nih.gov/sra) of the USA, China, the United Kingdom, Germany, Italy, Japan, Korea, the Netherlands, and Sweden, 206 metagenomes were obtained and analyzed (Supplementary Table S1).

### 2.2 Extraction of human DNA from metagenomic reads

In the present study, we defined the human gut metagenome as the data obtained by sequencing DNA samples extracted from feces. Human-derived DNA is present in feces and in the human gut metagenome. We searched for reads derived from human DNA in the human gut metagenome to eliminate glycan-related genes derived from human DNA. The FASTA files of human DNA were obtained from the NCBI database. Metagenomic reads were mapped to human DNA using BWA using this sequence as a reference (Li and Durbin, 2009). Reads mapped to human DNA were considered human-derived DNA from the stool samples and were excluded from all subsequent analyses.

### 2.3 Identification of glycan-related genes from human gut metagenomes

The amino acid sequence of the CAZyme was obtained from the dbCAN database (https://bcb.unl.edu./dbCAN2). Information on the enzyme classes of CAZymes, the species possessed, and enzyme function was obtained from CAZy (http://www.cazy.org). Approximately 810,000 sequences obtained by removing redundant sequences from the FASTA-format files of amino acid sequences of glycan-related genes from dbCAN were used as references for identifying other glycan-related genes. Alignments (Suzuki et al., 2014) between the amino acid sequences of the reference sequences and metagenomic reads were performed using GhostX. Glycan-related genes were identified according to the method of Takihara et al. (2021), and the metagenome read was considered a glycan-related gene and assigned the function of the gene according to the function of the DB sequences, showing an identity of 90% or more and an alignment length of 25 aa or more (Takihara et al., 2021). The genes GH, CE, and PL were assigned to the degradation group, the functional reads of GT were assigned to the synthesis group, and the relative abundance of each gene was calculated. The species of each read was the species derived from the assigned reference sequence.

### 2.4 Individual comparisons of the distribution of glycan-related genes

The conversion of enzyme function from the family was calculated by distributing the reads to enzymes within the CAZyme family and calculating the relative abundance. The Shannon index was calculated using *ad hoc* ruby scripts according to following equations:

$$H^{\prime }=-\sum_{i=l}^{S}P_{i}∙\log_{2}P_{i}\;\;\;S=Species\;counts,P_{i}=relative\;abundance$$

(Warwick et al., 2001).

Euclidean distances were determined, and hierarchical clustering was performed using the pheatmap library in R (https://cran.r-project.org/web/packages/pheatmap/index.html) to visualize the distribution of nationalities and enzyme functions.

### 2.5 Calculation of the distance between samples using *t*-distributed stochastic neighbor embedding (*t*-SNE)

Values of the relative abundance of enzymes classified as degradation enzymes were used to calculate and plot the distances between samples using *t*-SNE. The distance between samples was calculated using the Rtsne library (https://cran.r-project.org/web/packages/tsne/index.html) with default parameters (dims = 2, initial_dims = 50, perplexity = 30, theta = 0.5, max_iter = 1,000, momentum = 0.5, final_momentum = 0.8, eta = 200, exaggeration_factor = 12) and the distance between sample plots was calculated using the ggplot2 library (https://cran.r-project.org/web/packages/ggplot2/index.html).

### 2.6 Mapping to the kyoto encyclopedia of genes and genomes (KEGG) pathway

To investigate the roles of glycosyltransferases in the human gut environment, we mapped the EC numbers of transferases possessed by humans and those extracted from the gut metagenome to “Starch and sucrose metabolism” to the KEGG pathway (Kanehisa and Goto, 2000; Kanehisa, 2019; Kanehisa et al., 2021).

### 2.7 Substrates of genes with significant differences between clusters

The Wilcoxon rank-sum test was performed to determine the relative abundance of enzyme functions between clusters (*p*-value). The *q*-value was calculated using the BH method. The top 20 enzymes with the lowest *q*-values were selected. Enzyme substrates biased toward each cluster was classified as Plant, Animal, Mucin, Fungal, Peptidoglycan, Sucrose, and Starch, and the proportions between the clusters were compared. The enzyme substrates from multiple sources was distributed according to their abundance.

## 3 Results

### 3.1 Glycan-related genes identified from human gut metagenomes

**FIGURE 1:**
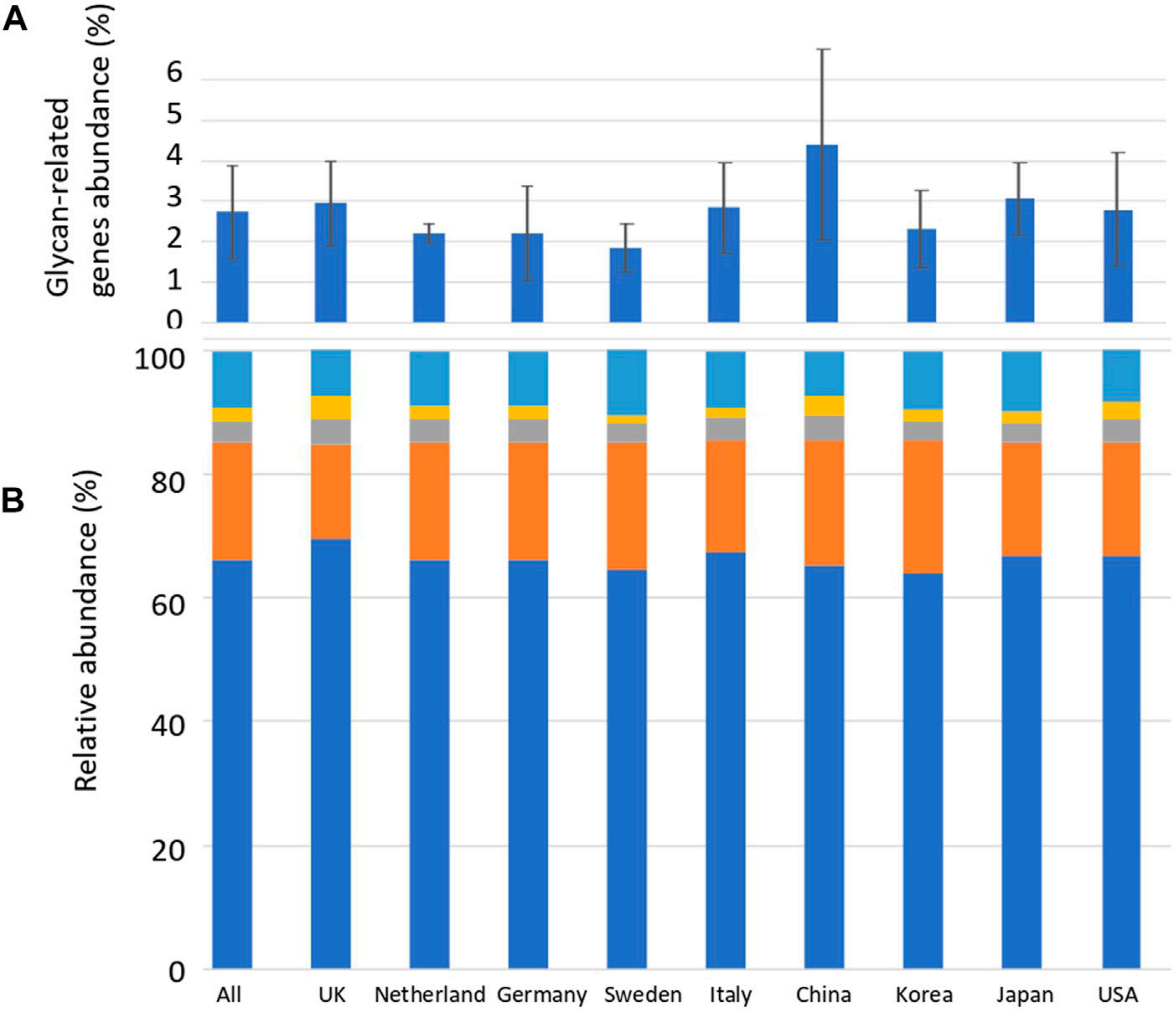
Average abundance of glycan-related genes and their relative abundance in CAZyme classification by country. **(A)** Percentage of glycan-related genes per total reads by nationality. Error bars represent the standard deviation. Countries are listed in order of longitude. **(B)** Average relative abundance of CAZy classification of the identified glycan-related genes by nationality. Blue: glycoside hydrolase (GH), Orange: glycosyltransferase (GT), Grey: carbohydrate esterase (CE), Yellow: polysaccharide lyase (PL), Right blue: carbohydrate-binding module (CBM).

Sequence alignment was performed between the reads of the human gut metagenome sequence and the CAZyme amino acid sequence downloaded from dbCAN. Reads exceeding the threshold of the calculated identity and alignment length were defined as glycan-related genes. The average number of carbohydrate-related genes per total read in the 207 human gut metagenomes was 2.8% (Figure 1; Supplementary Table S2). China_34 had the highest abundance (11.0%), and England_16 had the lowest abundance (0.64%) of glycan-related genes, showing a difference of more than 15 times (Figure 1; Supplementary Table S3). In this study, AA (auxiliary activities) had either no reads or only a few reads. Therefore, they were excluded from further analyses. The significance in the abundance of glycan-related genes between each country were calculated using Wilcoxon Rank-Sum test (Supplementary Table S4). Although there were significant differences between countries of less than 5%, no further comparisons were made due to differences in sample sizes across countries. The relative abundances of glycan-related genes classified by function were calculated and compared by nationality or as a whole. The relative abundance in 207 samples: 66.0% in GH, 19.0% in GT, 3.6% in CE, 2.3% in PL, and 8.8% in CBM (Supplementary Table S2). The significance in the relative abundance of gene function between each country were calculated using Wilcoxon Rank-Sum test (Supplementary Table S4). Although there were significant differences between countries of less than 5%, no further comparisons were made due to differences in sample sizes across countries.

### 3.2 Distribution of glycan-related genes other than those possessed by humans

**FIGURE 2:**
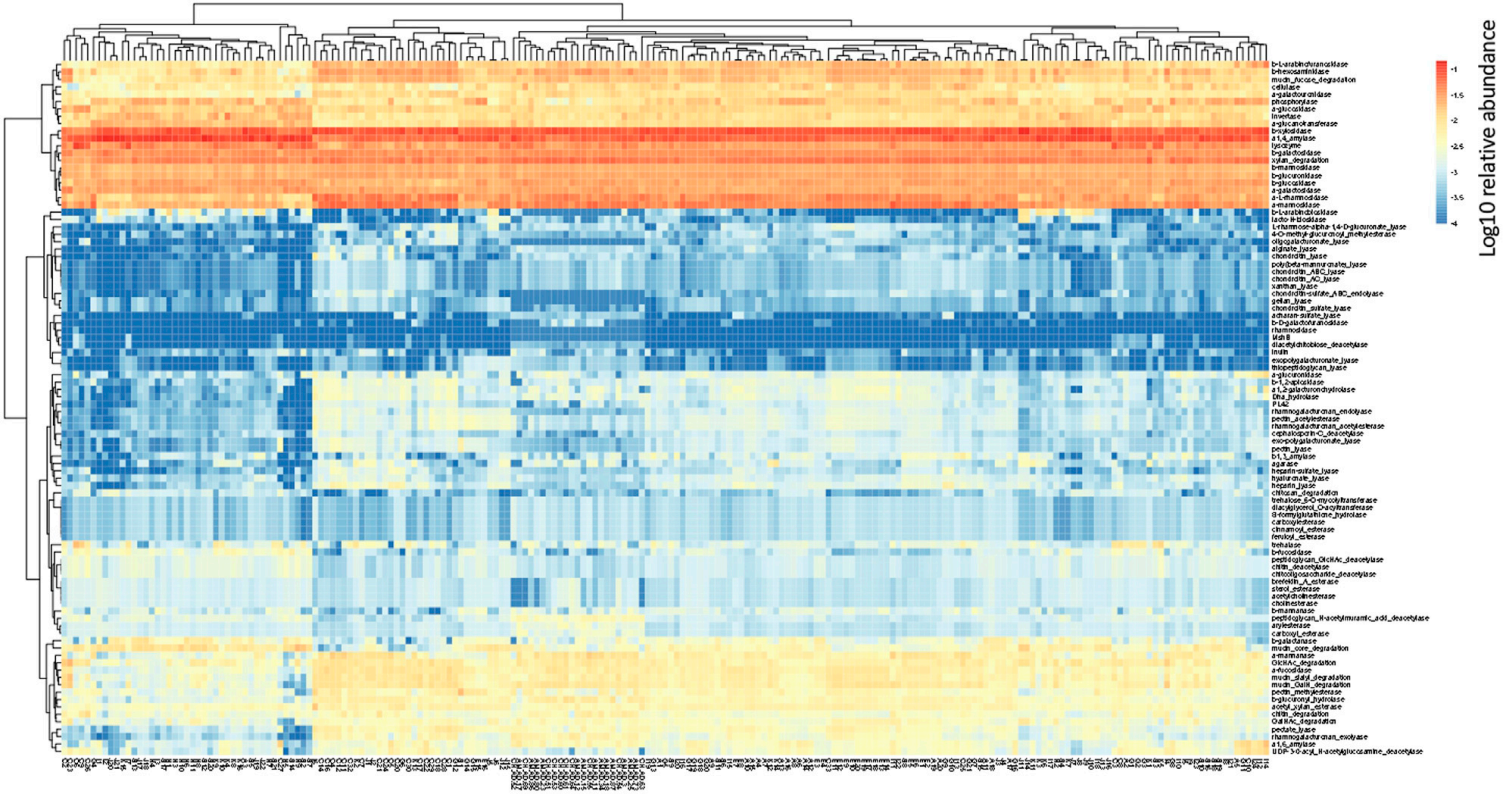
Clustering using the relative abundance of each enzyme function of GHs, CEs, and PLs classified as degradation. The name of the horizontal axis is described in Supplementary Table S1. Each value is the Log10 of the relative abundance of the gene.

**FIGURE 3:**
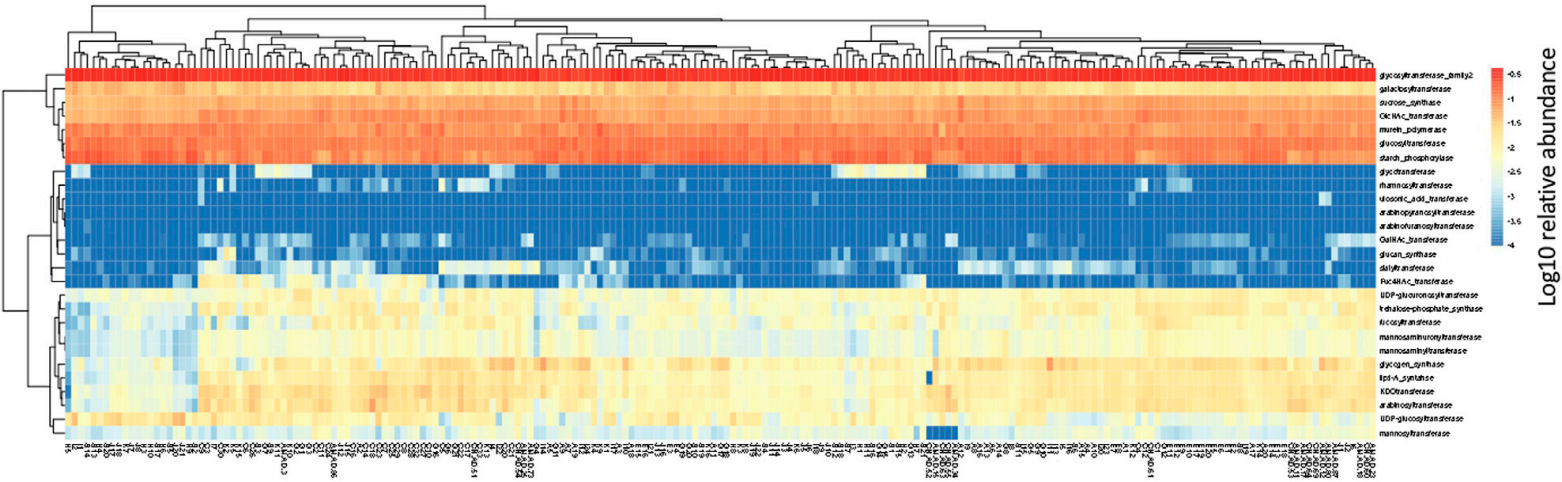
Clustering using the relative abundance of each enzyme function of GTs classified as synthesis. The name of the horizontal axis is described in Supplementary Table S1. Each value is the Log10 of the relative abundance of the gene.

In the human gut metagenome, 138 GH, 77 GT, 28 PL, and 14 CE families have been identified. Many amylases and lysozymes in GH, glucosyltransferases in GT, UDP 3-*O*-acyl N-acetylglucosamine deacetylases in CE, and pectate lyases in PL were detected (Figures 2, 3; Supplementary Tables S5, S6). Glycan-related genes perform two roles: carbohydrate degradation to break glycosidic bonds and glycosylation to form glycosidic bonds. Based on the enzyme function indicated by CAZy, the glycan-related genes were classified into “degradation” and “synthesis” types, and the ratio of each enzymatic function was calculated. The enzyme function of “synthesis” was similar among the samples, and nationality and individual differences were insignificant (Figure 3). However, there was a difference between the samples in terms of the type and abundance of the “degradation” enzyme function (Figure 2). Kaoutari et al. (2013) reported 17 human carbohydrate-degrading enzymes, all of which were detected in the present study (Supplementary Table S5). In addition to the enzymes present in humans, many other enzymes degrade plant-derived polysaccharides. Few enzymes were common in all samples, and others were either detected or not detected (Figures 2, 3; Supplementary Tables S5, S6).

### 3.3 Derivation of enzyme substrates enriched in the three clusters

**FIGURE 4:**
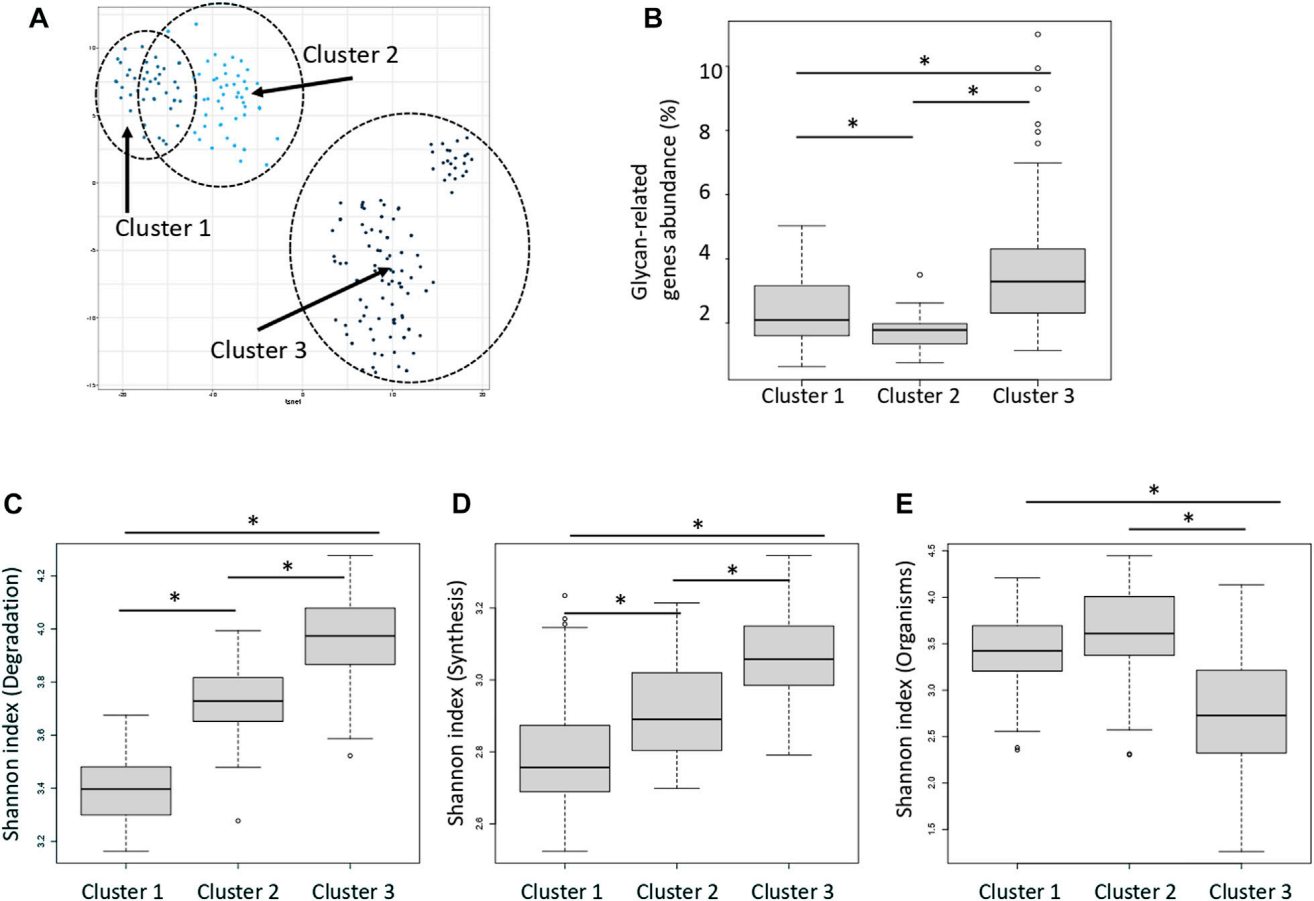
Three clusters classified by the relative abundance of carbohydrate-degrading enzymes. **(A)** Dot plot for three distinct clusters identified using the *t*-distributed stochastic neighbor embedding (*t*-SNE) algorithm based on the relative abundance of carbohydrate-degrading enzymes. Each dot represents each sample. Blue: cluster 1, Light blue: cluster 2, Dark blue: cluster 3. The eclipses show a 95% confidence. **(B)** Relative abundance of glycan-related gene in each cluster. *p < 0.01 compared using the Mann–Whitney test (*p* = 4.3E-4 between clusters 1 and 2; *p* = 3.8E-15 between clusters 1 and 3; *p* = 3.0E-5 between clusters 2 and 3). **(C)** Shannon index of the degradation genes in each cluster. *p < 0.01 compared using the Mann–Whitney test (*p* = 3.8E-14 between clusters 1 and 2; *p* = 3.7E-20 between clusters 1 and 3; *p* = 2.0E-13 between clusters 2 and 3). **(D)** Shannon index of the synthesis genes in each cluster. *p < 0.01 compared using the Mann–Whitney test (*p* = 5.1E-4 between clusters 1 and 2; *p* = 1.1E-10 between clusters 1 and 3; *p* = 1.2E-10 between clusters 2 and 3). **(E)** Shannon index of the organisms in each cluster. *p < 0.01 compared using the Mann–Whitney test (*p* = 2.7E-2 between clusters 1 and 2; *p* = 2.8E-11 between clusters 1 and 3; *p* = 1.0E-9 between clusters 2 and 3).

Distance between samples was calculated via *t*-SNE using the abundance and type of enzymes classified as “degradation” type (Figure 4A). The enzymes were classified into three groups according to the type of enzyme function detected in each sample and the distribution of their abundance. Figure 4A shows that the clusters are close to each other. These clusters were termed Clusters 1, 2, and 3. The average abundance of glycan-related genes in the three clusters was 1.8, 2.4, and 3.6%, respectively (Figure 4B; Supplementary Table S7). Cluster 3 had the highest abundance of glycan-related genes, whereas Cluster 1 had the lowest abundance and Cluster 2 was intermediate. No enzyme was significantly correlated to any country or region (Supplementary Figure S2; Supplementary Table S8). Major clusters were identified by country (Supplementary Figure S3). It is assumed that this was caused by the difference in food culture, because there were differences between Europe and Asia. In the three clusters, the Shannon index was calculated using three patterns; degradation genes, synthesis genes, organisms. The Shannon index of the three clusters were compared to compare the diversity of each cluster. Cluster 3 had a high value calculated as the abundance of genes for the degradation and synthesis, and cluster 1 had a low value (Figures 4C, D; Supplementary Table S9). On the other hand, when comparing the Shannon index calculated by the abundance of species possessed, index of cluster 1 was high and index of cluster 3 was low (Figure 4E).

**FIGURE 5:**
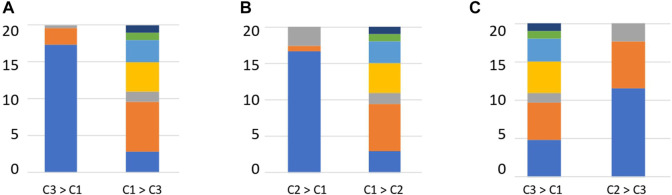
Percentage of substrates of enzymes that are significantly different between clusters. **(A)** Enzyme substrates significantly different between clusters 3 and 1. **(B)** Enzyme substrates significantly different between clusters 2 and 1. **(C)** Enzyme substrates significantly different between clusters 3 and 2. Blue: Plant, Orange: Animal, Grey: Mucin, Yellow: Fungal, Right blue: Peptidoglycan, Green: Sucrose, Deep blue: Starch.

The relative abundance of each enzyme function was compared between clusters to determine the enzyme functions involved in cluster formation. Enzymes, such as DHA hydrolases and PLs, which can degrade plant-derived polysaccharides, were obtained (Supplementary Table S10). We investigated the substrates of the top 20 enzymes based on the order of the calculated significant differences. Clusters 2 and 3 contained many enzymes that degrade plant-derived polysaccharides (Figure 5). In contrast, in cluster 1, many enzymes degraded animal-derived polysaccharides, mucins, peptidoglycans, and fungus-derived polysaccharides (Figure 5). These results demonstrated that the clusters classified by the type and abundance of carbohydrate-degrading enzymes had different degradation potentials depending on the type of decomposing substrate.

### 3.4 Species enriched in the three clusters

**FIGURE 6:**
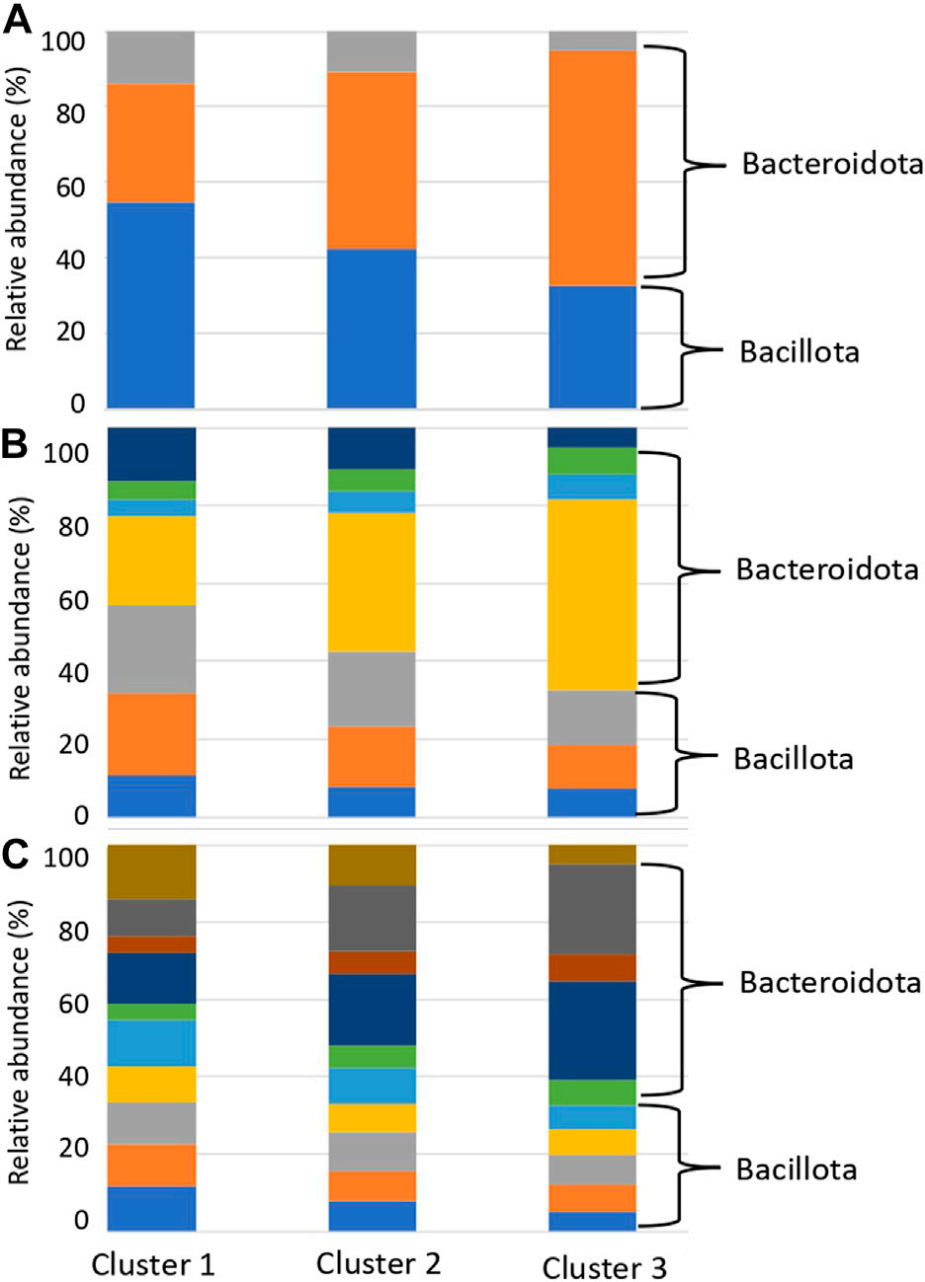
Relative abundance of species with glycan-related genes in each cluster. **(A)** Phylum level. Blue: Bacillota, Orange: Bacteroidota, Grey: Actinomyceota. **(B)** Family level. Blue: Eubacteriaceae, Orange: Lachnospiraceae, Gray: Oscillospiraceae, Yellow: Bacteroidaceae, Light blue: Rikenellaceae, Green: Tanerellaceae, Deep blue: Bifdobacteriaceae. **(C)** Genus level. Blue: *Blautia*, Orange: *Eubacterium*, Gray: *Faecalibacterium*, Yellow: *Roseburia,* Light blue: *Ruminococcus*, Green: *Alistipes*, Deep blue: *Bacteroides*, Brown: *Parabacteroides*, Ocher: *Phocaeicola,* Dark brown: *Bifidobacterium*.

We presumed that the organism species from which the genes with reference sequences in the sequence alignment were derived were the organism species of the identified glycan-related genes, and compared the distribution of organism species by country and cluster. Compared to clusters 1 and 2, cluster 3 had a higher ratio of matching enzymes from bacteria belonging to Bacteroidota, consisting of *Bacteroides* and *Phocaeicola* (Figure 6; Supplementary Table S11). In contrast, Cluster 1 had a high ratio of matching enzymes of microorganisms belonging to Bacillota, consisting of *Blautia*, *Faecalibacterium*, and *Bifidobacterium*. *Bacteroides*, *Phocaeicola* and *Parabacteroides* were more abundant in cluster 3 than in clusters 1 and 2 (Supplementary Table S12). *Bifidobacterium*, *Eubacterium, Ruminococcus, Roseburia* and *Blautia* were more abundant in cluster 1 than in clusters 2 and 3 (Supplementary Table S12). *Faecalibacterium* was more abundant in Cluster 2, although the difference was not significant.

## 4 Discussion

Interactions between humans and the gut microbiome occur by supplying nutrients to gut epithelial cells via short-chain fatty acids obtained from dietary carbohydrates or mucins and activating immunity via mucin degradation (Hasain et al., 2020). The degradation distribution revealed the presence of clusters rich in plant-derived polysaccharides and non-plant-derived polysaccharides (Figure 5). *Bacteroides fragilis* possesses many glycolytic enzymes, including 155 GHs, and the Bacteroidota group has been suggested to play a role in the degradation of polysaccharides in the human gut environment (Kaoutari et al., 2013). The *Bacteroides* species has been reported to be capable of degrading various polysaccharides and oligosaccharides (Ndeh et al., 2018; Lapébie et al., 2019). Degradation of mucins present on or secreted from the surface of gut epithelial cells is primarily performed by *Bacteroides* (Desai et al., 2016; Luis et al., 2021), *Akkermansia* (Everard et al., 2013) and *Bifidobacterium* (Katayama et al., 2004). *Bifidobacterium* has two types of enzymes: an enzyme that cuts sialic acid at the end of mucin, and an enzyme that cleaves GalNAc of mucin peptides, and is considered an important species that provides carbohydrates via mucin degradation in the human gut environment (Fujita et al., 2005). *Ruminococcus* species have been reported to have the ability to degrade resistant starch (Ze et al., 2012; Ze et al., 2015; Mukhopadhya et al., 2018).

There is a correlation between carbohydrate degradation and the type of microorganisms in the gut, which is suggested to play a role in controlling the degradation of polysaccharides and the abundance and composition of degradation products in the human gut environment. Species that can adapt to the type and abundance of dietary carbohydrates are responsible for the initial stage of polysaccharide degradation, whereas species that produce short-chain fatty acids from degraded carbohydrates dominate the later stages (Coyte and Rakoff-Nahoum, 2019). Carbohydrates are thought to be distributed throughout the gut microbiome, and the fatty acids they produce are the basis for interactions between humans and the gut microbiome.

Glycogen, peptidoglycan, and polysaccharide synthesis, such as β-glucan synthesis, were observed (Figure 3; Supplementary Table S6). Transferases identified for starch and sucrose metabolism in the KEGG pathway between humans and the gut microbiome were mapped to the polysaccharide synthesis pathway (Supplementary Figure S4). It has been suggested that transferases in the gut microbiome store carbohydrates by synthesizing polysaccharides, which may eventually regulate the rate at which humans absorb monosaccharides. Altered carbohydrate chains have been detected in the gut epithelial cells of patients with inflammatory bowel disease but not in healthy individuals (Kudelka et al., 2020). Inflammatory bowel disease is thought to be caused by an imbalance in the gut microbiome, which subsequently affects the onset and exacerbation of the disease (Walker et al., 2011; Smits et al., 2017). Mice lacking GGTA, a gene that synthesizes α-Gal, show an altered gut microbiome and immune responses (Singh et al., 2021a; Singh et al., 2021b).

There is a glycan-mediated interaction between the host and microbiome, and the formation of clusters by glycan synthesis genes was less pronounced than that by degradation (Figure 3). In the genes of the degradation of human gut bacteria, 1) classified into 3 groups, although the proportion of each group is different for each country, 2) each group shows a different diversity index, 3) there are different species between groups, and enzymes with different substrates origins. Based on these findings, the following two points were predicted:1) diversity will be constant because the influence on the host side by the transferase of gut bacteria is a function derived from the genome, and 2) diversity will be high because it is affected by incoming dietary carbohydrates. The Shannon index showed different tendency when calculated using the relative abundance of genes and bacterial species. A small number of species are expected to have multiple genes.

The degradation clusters were more individual than national clusters (Supplementary Figure S3). Dietary carbohydrates have been reported to alter gut microbiota (Cresswell et al., 2020). The composition of glycan-related genes possessed by the gut microbiota may be largely determined by individual habits and innate factors rather than geographical and cultural factors. However, major clusters were observed in each country (Supplementary Figure S3). Although the influence of individual lifestyle and culture was expected, no further detailed analysis was possible because dietary information for individual samples was unavailable.

This phenomenon, in which the gut microbiota composition is derived from individual lifestyles rather than geographical influences, is consistent with the *16S rRNA* enterotype (Arumugam et al., 2011; Wu et al., 2011). In the clusters formed by the degradation patterns, differences were observed in *Bacteroides* and *Ruminococcus*, which are the major constituents of the *16S rRNA* enterotype (Figure 6; Supplementary Table S11). Kaoutari et al. reported that *Bacteroides* play a role in degrading plant-derived polysaccharides, while *Ruminococcus* plays a role in degrading other polysaccharides (Kaoutari et al., 2013). *Prevotella* did not differ among the degradation clusters but was thought to play other non-carbohydrate-related roles. The distribution of carbohydrate degradation and addition potential of the gut microbiota demonstrated that the composition of the gut microbiota has parts with high diversity and parts with low diversity. In addition, these compositions may be influenced more by dietary carbohydrates than geographical factors.

These things have not yet been clarified because the focus has been on genes with high abundance or those that exhibit significant differences. Differences between perspectives can be determined by comparing genes using a database in which the genes are finely classified by function and species. For example, examining the degradation potential of oligosaccharides used in personal prebiotics and constructing a baseline indicating the degree of health of the gut microbiome can aid in developing personalized medicine. Bamgbose et al., 2022, Modesto et al., 2023.

## Data Availability

The datasets presented in this study can be found in online repositories. The names of the repository/repositories and accession number(s) can be found in the article/Supplementary Material.
