# Supplementary figures and images for: Glycan-related genes in human gut microbiota exhibit differential distribution and diversity in carbohydrate degradation and glycan synthesis
Source: Front Mol Biosci. 2023 Jun 15;10:1137303. doi: 10.3389/fmolb.2023.1137303 (PMC10311216; doi:10.3389/fmolb.2023.1137303)

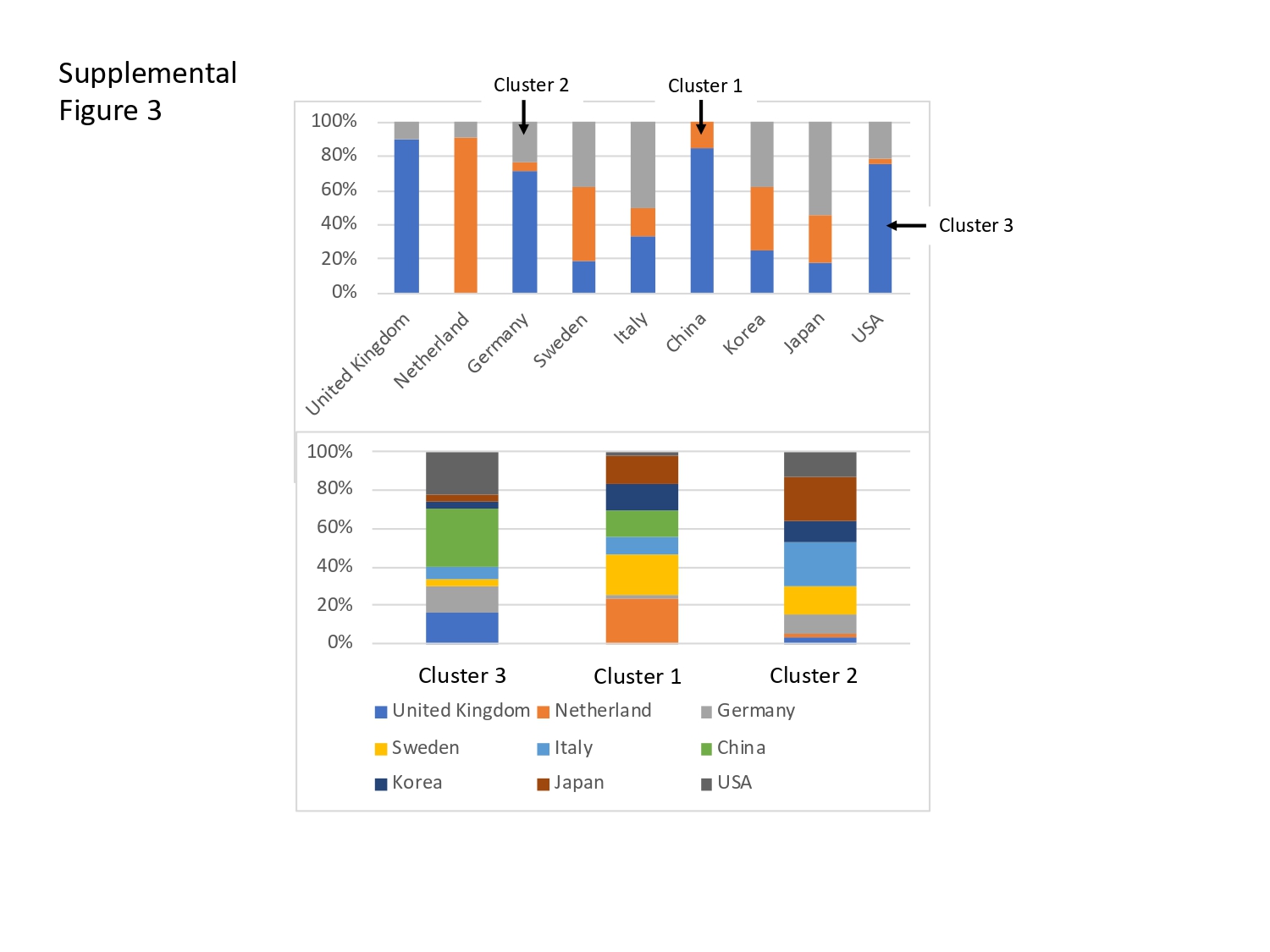

Supplement: Supplementary file 1 [file Image3.JPEG]

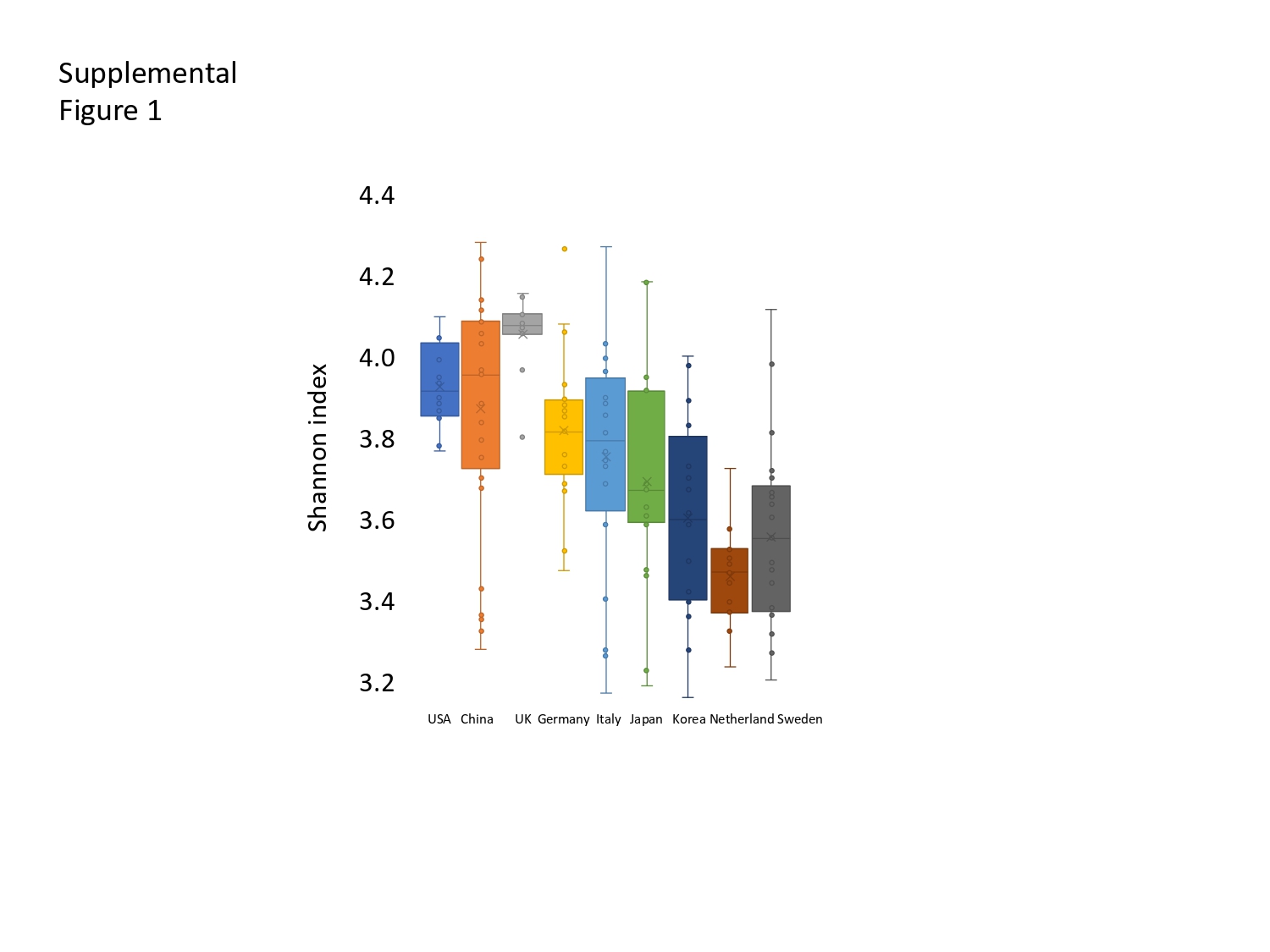

Supplement: Supplementary file 2 [file Image1.JPEG]

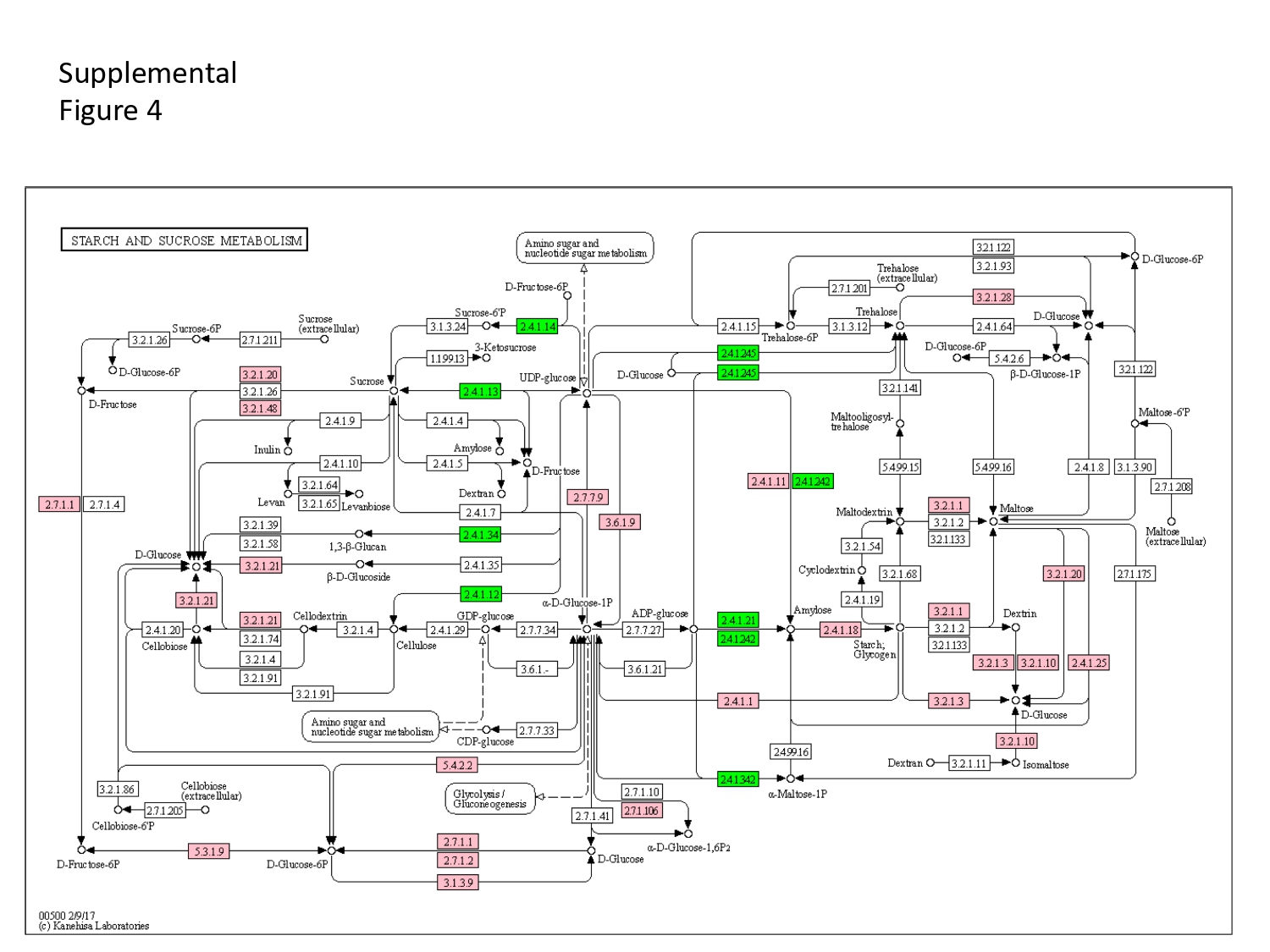

Supplement: Supplementary file 3 [file Image4.JPEG]

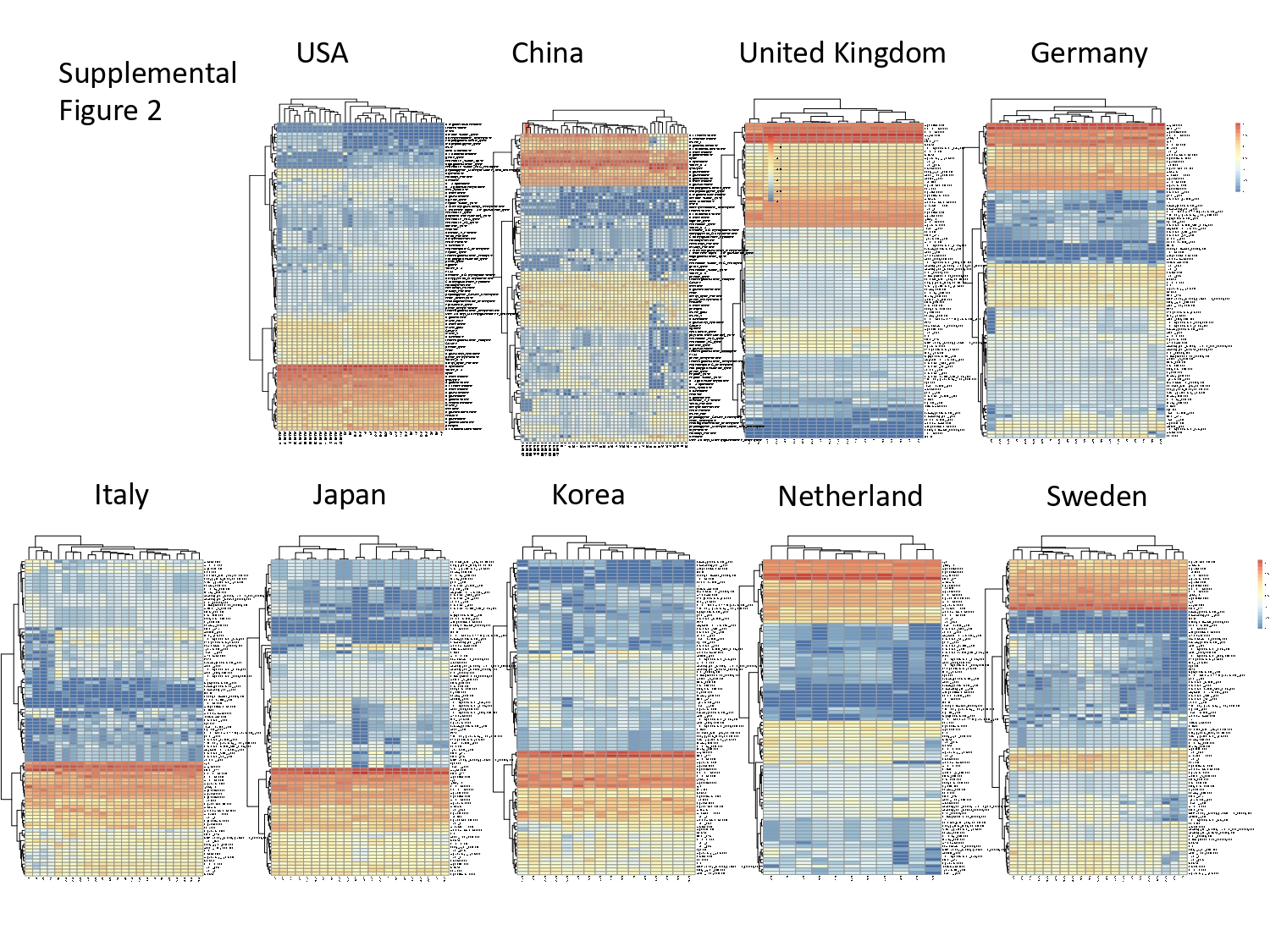

Supplement: Supplementary file 4 [file Image2.JPEG]
