# Supplementary material for: Glycan-related genes in human gut microbiota exhibit differential distribution and diversity in carbohydrate degradation and glycan synthesis
Source: Front Mol Biosci. 2023 Jun 15;10:1137303. doi: 10.3389/fmolb.2023.1137303 (PMC10311216; doi:10.3389/fmolb.2023.1137303)
Supplement: Supplementary file 5 [file Table2.DOCX]

Supplementary Material

# Supplementary Material

## Supplementary Figures

**Supplementary Figure 1.** Box plot of Shannon index calculated using the relative abundance of genes in each sample.

**Supplementary Figure 2.** Clustering by country using the relative abundance of each enzyme function of glycoside hydrolases (GHs), carbohydrate esterases (CEs), and polysaccharide lyases (PLs) was classified as degradation.

**Supplementary Figure 3.** Percentage of the three clusters by country. Orange : cluster 1, Gray : cluster 2, Blue : cluster 3.

**Supplementary Figure 4.** Mapping of synthesis enzymes to starch and sucrose metabolism in the Kyoto Encyclopedia of Genes and Genomes (KEGG) pathway. Red: Human, Green: Gut microbiota.

## Supplementary Tables

**Supplementary Table 1.** List of metagenomic samples used in this study.

**Supplementary Table 2.** Average of relative abundance of glycan-related genes and relative abundance by CAZyme function by nationality.

**Supplementary Table 3.** Glycan-related genes identified in each metagenomic sample.

**Supplementary Table 4.** Significance of gene abundance, relative abundance of GH, GT, CE, PL, CBM.

**Supplementary Table 5.** Relative abundance of carbohydrate-degrading enzymes.

**Supplementary Table 6.** Relative abundance of glycan synthesis enzymes.

**Supplementary Table 7.** Relative abundance of glycan-related genes per sample for three clusters.

**Supplementary Table 8.** Percentage of three clusters by country.

**Supplementary Table 9.** Shannon index of the degradation genes, the synthesis genes and organisms.

**Supplementary Table 10.** Significant differences in genes between clusters and derivates of enzyme substrates.

**Supplementary Table 11.** Relative abundance of organisms with glycan-related genes.

**Supplementary Table 12.** Significant differences in bacteria species between clusters.
